# Spotted Fever

**Published:** 1863-10

**Authors:** E. W. Jenks

**Affiliations:** Sturgis, Mich.


BUFFALO

### Medical and Surgical Journal.

VOL. III. OCTOBER, 1863. NO. 3

### ORIGINAL COMMUNICATIONS.

ART. I.—*Spotted Fever*—By E. W. Jenks, M. D., *Sturgis, Mich.*

During the last winter and spring a new form of disease has prevailed in different parts of the country, which has by common consent received the name of "spotted fever." In this immediate vicinity I saw no cases; ten miles from here, in La Grange county, Ind., the disease prevailed to quite an extent; a number of cases proving suddenly fatal. In other portions of Northern Indiana the disease was more common and still more fatal.

My own preconceived ideas of the disease, from what I had heard, were that it was a variety of typhus. The first case I saw perplexed me exceedingly, and I was ready to call it either typhus or cerebro spinal meningitis. From repeated observation I am convinced that it was neither one or the other, but a distinct and peculiar disease, having its own peculiar morbific cause and phenomena.

Without giving a history of each individual case, I will give only the general characteristics of the disease, as it presented itself to me; among the most striking of which, were the suddenness of attack, and in fatal cases, the sudden fatality. Most of the patients were attacked with a chill following which would be the sudden occurrence of headache, mostly in posterior region of the head, with severe spinal pain, sometimes extending to the limbs. Soreness of the flesh of all parts of the body was complained of in almost every instance, so as to elicit tokens of suffering whenever the patient was moved, even in those cases where there appeared to be almost complete stupor. In the majority of cases the head was drawn back, and no proper amount of force could bring the chin to the breast. Immediately following these symptoms, and in some cases simultaneous with them, was the characteristic eruption, which was of a dark purple color, non-elevated, and not receding upon pressure; there would also be some lighter colored spots, making a gradation of color from the dark ecchymoid spots, to those of a light red. There was no uniformity in the size of these spots. some were not larger than a pin's point, while some were one-half to three-fourths of an inch in diameter. In one case I saw, in addition to the spots I have described, several large elevated spots, of the size of a twenty-five cent piece, of very dark color, presenting outside of the dark color a blistered appearance. Dr. Fletcher, of Lima, Ind., informed me that in several instances he observed these elevated blistered spots.

There was sometimes vomiting in the commencement of the attack, with an abhorrence of food. I neither observed or heard of any case of diarrhœa or abdominal tenderness; in every case there was obstinate constipation. The febrile symptoms varied, in the sudden fatal cases none followed the chill, but the pulse was feeble and the skin cold. In none of the cases was there a strong, full pulse, and the heat of the surface was less in all cases than is usually observed in acute diseases. Dr. George Fletcher, of Lima, Ind., with whom I saw some cases of this disease, and to whom I am indebted for an account of some of his observations, says that in one case which recovered, the patient lost permanently the use of one eye, there being complete amaurosis. In another case there was strabismus and curvature of the spine, which continued at last accounts, several months after. In one fatal case I saw, there was swelling of cervical and sub-maxillary glands. There was not complete delirium in any case, the tendency was more to stupor than delirium; the patients could usually be aroused so as to give intelligent answers to questions; in all fatal cases the patients died comatose.

In one case only was I permitted a post-mortem examination. The patient, a girl aged 13, went to bed at night apparently well; getting up in the night to obtain a drink of water she suddenly lost the use of her limbs. Her parents not hearing her return to bed, got up and found her on the floor; she said she could not walk, and complained of cold, headache, and soreness of limbs. I saw her the next morning; she was lying with her head thrown back, the surface of the body was cold, and covered with the characteristic symptoms; the pulse was slow and feeble, the pupils were dilated, the bowels were neither distended or tender.—She was in a state of stupor, yet when aroused would complain of severe pain in head, back and limbs. The next day there were more febrile symptoms, yet at no time as manifest as is usual in acute diseases. In this case only did I see any glandular swellings about the neck. She remained in about the same condition until the third day, and then died comatose. Autopsy was made twelve hours after death. The brain was found very much congested, the veins being distended to their utmost capacity; there was a small amount of serum effused at the base of the brain and there appeared to be a slightly softened condition of the upper portion of the spinal cord. The left cavities of the heart were entirely empty, while those of the right side were filled with very dark colored blood, with small amount of coagula. The dependent portions of the lungs only were congested, otherwise they had a healthy appearance. I regret that I was not allowed time to examine the abdominal viscera.

Without giving the details of treatment in any of the cases of "spotted fever," I would merely say that the treatment most successful, was upon the sustaining plan, viz: brandy, quinine, beef tea, and tinct. ferri mur.

The mortality of spotted fever was very great; in the majority of fatal cases they were speedily fatal; the commencement of the attack was the time to be watchful; those patients that lived several days were quite apt to recover, although recovery was very slow.

The nomenclature and pathology of this disease have been subjects of much disputation. Many medical men have denied the existence of such a disease. Some have called it by one name, and some by another. I have been unable to find in any medical work a description of the disease as it presented itself to my notice. I do not find it mentioned in any work on the Practice of Medicine. Dr. Bartlett, in his work on "Fevers in the United States," speaks of a disease which prevailed in New England between the years of 1807 and 1815, which was commonly called spotted fever and was supposed by some writers to be the true typhus. Dr. B. says, "It is very certain that in many important particulars it bore a striking resemblance to true typhus. This resemblance is noticed by most writers upon the disease. Dr. Elisha North called it *a new petechial typhus*. Dr. Hale of Boston, regards the disease as it prevailed in Gardiner, Maine, in 1814, as a congestive fever." "It is not easy," says Dr. B., "at the present day, upon such evidence as we possess, to decide with any confidence upon the precise character of the spotted fever of New England." Dr. Bartlett's conclusions of the spotted fever of New England, from all information he could obtain of the disease, were such as might be proper to conclude of the spotted fever of this vicinity, viz: "that it belongs to that class of new and more or less temporary epidemics, each having its peculiar character, marked by its peculiar phenomena, and depending upon new and peculiar combination of unknown morbific influences, which have always from time to time made their appearance, rather than to the class of established and permanent maladies."

While engaged in writing this article, my attention has been called to quite an elaborate paper upon "Spotted Fever as seen in the vicinity of Philadelphia in 1853," by Dr. W. W. Gerhard, included among the "Transactions of the College of Physicians of Philadelphia," and published in "The American Journal of Medical Science," for July, 1853. I am extremely gratified that my own views and limited observations of the disease correspond so closely with such high authority. I would commend the paper to all who wish to investigate this disease, but more especially to those who have questioned its existence, this paper coming from such an authoritative source will help dispel all such doubts.

This paper of Dr. Gerhard's is the first I have ever seen in print describing "spotted fever," so as to correspond with the disease as it prevailed in this vicinity. My own views of the pathology, diagnosis, and nomenclature of the disease are given in this paper, and since the publication of it I can add nothing more appropriate than is given in the language of Dr. Gerhard. He says, "Although the proof of spotted fever being a "blood disease, is to my mind conclusive, it must not be ascribed to an im- "poverished condition of this fluid from innutritious or deficient food, as "none of the patients whom I saw were in a condition of actual poverty, "and a large majority belonged to a class amply supplied with all the com- "forts of life."

"The disease indeed, is one which I should place in the list of rare "peculiar disorders, evidently depending upon diseased conditions of the "blood, which occasionally show themselves; they last for a time, then dis- "appear, but are not sufficiently permanent in their attacks to find a place "in the regular treatises on the practice of medicine. The diagnosis "becomes easy to one who has become familiar with the disease, from the "individual physiognomy of the patient. There is a peculiar dusky hue,

"and an expression of stupor conjoined with the eruption which character-"ize the disease at once, especially if we add to these signs the cerebral "symptoms of the disease.

"Inasmuch as this disease is attended by no definite anatomical lesions, "the appellation given to it more than half a century ago by Dr. Gallup, "and others in New England, of spotted fever, should be retained. It is "sufficiently characteristic and involves no doubtful point in question. The "only objection to the term is that the disease may be confounded with "epidemic typhus or ship fever, in which the whole body is also covered "with spots, but these constitute a real exanthema, and are, of course of a "totally different character and aspect from the eruption of spotted fever. "The latter is little else than a hemorrhagic effusion, very like that of "scurvy

"The most important remedies are stimulants. The necessity for stimu-"lation is based upon the rapid loss of force which takes place in this dis-"ease. The disease I believe to be produced by some unknown cause, "which we may call poison, if we choose, acting upon the body. A certain "time is required for the elimination of it from the system, and during "this time we must support the strength by appropriate food and stim-"ulants."

